# Optical Quantal Analysis Using Ca^2+^ Indicators: A Robust Method for Assessing Transmitter Release Probability at Excitatory Synapses by Imaging Single Glutamate Release Events

**DOI:** 10.3389/fnsyn.2019.00005

**Published:** 2019-03-04

**Authors:** Zahid Padamsey, Rudi Tong, Nigel Emptage

**Affiliations:** ^1^Center for Discovery Brain Sciences, University of Edinburgh, Edinburgh, United Kingdom; ^2^Department of Pharmacology, University of Oxford, Oxford, United Kingdom

**Keywords:** optical quantal analysis, Ca^2+^ imaging, presynaptic plasticity, hippocampus, Schaffer-collateral, release probability

## Abstract

Despite evidence that presynaptic efficacy and plasticity influence circuit function and behavior *in vivo*, studies of presynaptic function remain challenging owing to the difficulty of assessing transmitter release in intact tissue. Electrophysiological analyses of transmitter release are indirect and cannot readily resolve basic presynaptic parameters, most notably transmitter release probability (*p*_r_), at single synapses. These issues can be circumvented by optical quantal analysis, which uses the all-or-none optical detection of transmitter release in order to calculate *p*_r_. Over the past two decades, we and others have successfully demonstrated that Ca^2+^ indicators can be strategically implemented to perform optical quantal analysis at single glutamatergic synapses in *ex vivo* and *in vitro* preparations. We have found that high affinity Ca^2+^ indicators can reliably detect spine Ca^2+^ influx generated by single quanta of glutamate, thereby enabling precise calculation of *p_r_* at single synapses. Importantly, we have shown this method to be robust to changes in postsynaptic efficacy, and to be sensitive to activity-dependent presynaptic changes at central synapses following the induction of long-term potentiation (LTP) and long-term depression (LTD). In this report, we describe how to use Ca^2+^-sensitive dyes to perform optical quantal analysis at single synapses in hippocampal slice preparations. The general technique we describe here can be applied to other glutamatergic synapses and can be used with other reporters of glutamate release, including recently improved genetically encoded Ca^2+^ and glutamate sensors. With ongoing developments in imaging techniques and genetically encoded probes, optical quantal analysis is a promising strategy for assessing presynaptic function and plasticity *in vivo*.

## Introduction

The efficacy of synaptic input critically shapes circuit function and behavior. Synaptic efficacy is comprised of two main features: (1) postsynaptic potency (*q*), which refers to the amount of postsynaptic depolarization generated by a synapse in response to a single quantum of neurotransmitter and (2) presynaptic release probability (*p_r_*), which refers to the likelihood a synapse will release a single quantum of neurotransmitter. Both pre- and postsynaptic efficacy impact cellular and circuit operations (Evans et al., 2018; Grillo et al., 2018), as well as undergo activity-dependent changes *in vivo* (Maren, 2005; Koga et al., 2015; Choi et al., 2018). Whereas several techniques can be employed to measure *q*, examination of *p_r_* is often more challenging owing to the difficulties of assessing transmitter release in intact tissue. Although there are a number of electrophysiological approaches that can be employed to assess presynaptic efficacy, they are indirect and cannot readily resolve presynaptic release at single synapses (see Clements and Silver, 2000; Yang and Calakos, 2013 for a review of electrophysiological approaches to quantal analyses and their limitations). Such issues can be resolved by optical quantal analysis.

Optical quantal analysis is a means of assessing *p_r_* based on the all-or-none optical detection of transmitter release. In principle this can be done with any fluorescent reporter of vesicular fusion or transmitter release, and at any synapse (see “Discussion”). The fluorescent reporter used for detecting transmitter release, however, must afford sufficient sensitivity to reliably detect quantal release at the chosen synapse, and in the chosen experimental preparation. Over the past two decades we have demonstrated that Ca^2+^ indicators provides one such means for robustly assessing transmitter release at glutamatergic synapses in *in vitro* and *ex vivo* slice preparations by enabling the detection of all-or-none, excitatory synaptically evoked postsynaptic Ca^2+^ transients [EPSCaTs; (Emptage et al., 1999)].

**FIGURE 1:**
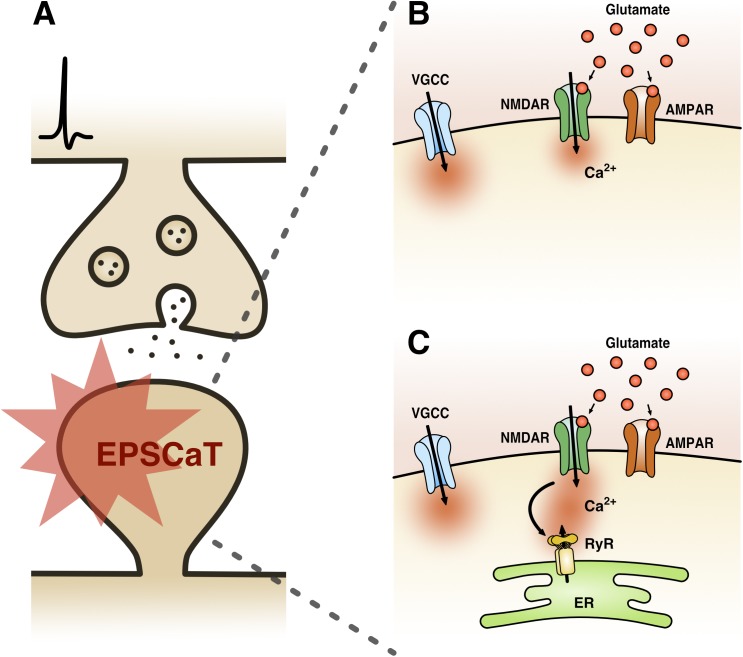
Excitatory postsynaptic Ca^2+^ transient (EPSCaT) pharmacology. **(A)** Schematic of an EPSCaT-generating synapse responding to uniquantal glutamate release. **(B)** EPSCaT signals are derived from NMDAR- and VGCC-mediated Ca^2+^ influx, driven by AMPAR-mediated depolarization. **(C)** At synapses containing ER, EPSCaTs are additionally and reliably amplified by Ca^2+^ induced Ca^2+^ release (CICR), which is triggered by NMDARs and mediated by RyRs. In some instances, EPSCaTs will also show a delayed IP_3_R-mediated Ca^2+^ component, triggered by group 1 metabotropic glutamate receptors (mGluRs) signaling (not shown).

EPSCaTs are present at most central glutamatergic synapses [including hippocampal, neocortical, striatal, and amygdalar (Bloodgood and Sabatini, 2007)], though have been most extensively studied at hippocampal Schaffer-collateral synapses (Figure 1). Here, it has been well established that a single quantum of glutamate can trigger sufficient Ca^2+^ influx into a dendritic spine that can be detected by high affinity Ca^2+^ indicator dyes, such as Oregon Green BAPTA-1 (OGB-1) (Emptage et al., 1999). This Ca^2+^ influx is mediated by both NMDA receptors (NMDARs) and voltage-gated Ca^2+^ channels (VGCCs), activation of which is driven by AMPA receptor (AMPAR)-mediated depolarization (Ngo-Anh et al., 2005; Grunditz et al., 2008; Padamsey et al., 2017) (Figure 1B). The recruitment of these Ca^2+^ sources by uniquantal glutamate release likely reflects the strong electrical compartmentalization of the spine head (Harnett et al., 2012; Beaulieu-Laroche and Harnett, 2018). In spines containing endoplasmic reticulum (ER), which comprise approximately 10–20% of Schaffer collateral synapses, additional Ca^2+^ release is triggered reliably via RyR-gated stores, and also via IP_3_R-gated stores, albeit with delayed kinetics and reduced probability (Emptage et al., 1999;Holbro et al., 2009; Padamsey et al., 2018) (Figure 1C). Because release of single quanta of glutamate can drive detectable levels of Ca^2+^ influx in dendritic spines, the probability of eliciting an EPSCaT with single presynaptic stimuli can be used as a proxy for *p_r_*. Indeed, we have shown that the probability of evoking EPSCaTs is physiologically and pharmacologically similar to *p_r_*: (1) both are stochastic all or none-events (Emptage et al., 1999) that (2) have similar means and distributions (Ward et al., 2006), and (3) similarly exhibit short-term facilitation (Emptage et al., 1999, 2003). Moreover, (4) EPSCaT probability, like *p_r_*, scales with the size of the active zone (Holderith et al., 2012) and (5) can be decreased by baclofen and adenosine, which are known to decrease *p_r_* (Emptage et al., 1999; Oertner et al., 2002;Chalifoux and Carter, 2010).

In this paper, we provide a detailed protocol for using Ca^2+^ dyes to measure *p_r_* at Schaffer-collateral synapses in hippocampal slices. We describe extensions and applications of the technique, including its use for assessing activity-dependent presynaptic plasticity. The general technique we describe here can be used at other synapses, and with other sensors of glutamate release, including genetically encoded Ca^2+^ and glutamate sensors. We end by discussing the limitations of our method, and highlight the future potential of optical quantal analysis, especially in light of recent advancements in genetically encoded sensors and *in vivo* imaging techniques.

## Materials and Methods

We begin with the core technique, which involves (1) preparing brain slices, (2) loading a postsynaptic cell with Ca^2+^ indicator dye, (3) searching for a spine that generates EPSCaTs in response to electrical stimulation, and (4) calculating *p_r_* from these responses.

### Optical Quantal Analysis With Ca^2+^ Indicators

#### Brain Slice Preparation

In our labs, we focus on synaptic properties at CA3-CA1 synapses assessed in either acute or cultured hippocampal slices, which are prepared according to previously published methods (Emptage et al., 2003; Padamsey et al., 2017). Both acute and cultured slices have been shown to have very similar synaptic properties and forms of plasticity (De Simoni et al., 2003; Enoki et al., 2009; Padamsey et al., 2017). For acute slice preparation, coronal acute hippocampal slices (400 μm) are made from the brains of 2–3 week old male Wistar rats. Dissection and slice preparation is performed in an ice cold, sucrose-based ACSF solution (in mM: 85 NaCl, 65 sucrose, 26 NaHCO_3_, 10 glucose, 7 MgCl_2_, 2.5 KCl, 1.2 NaH_2_PO_4_, and 0.5 CaCl_2_) that is bubbled with 95% O_2_ and 5% CO_2_. Slices are allowed to recover at room temperature for at least 1 h in normal ACSF (in mM: 120 NaCl, 2.5 KCl, 26 NaHCO_3_, 11 glucose, 1 MgCl_2_ 1.2 NaH_2_PO_4_, and 2 CaCl_2_) prior to recording. Because of the thermal sensitivity of presynaptic release, recordings are done at near physiological temperatures 32–33°C (Pyott and Rosenmund, 2002).

Cultured hippocampal slices (350 μm), which offer excellent optical access to subcellular compartments, are prepared from male Wistar rats (P7–P8). Brains are dissected in ice cold Earle’s Balanced Salt Solution (EBSS) with added glucose (+35 mM) and HEPES (+20 mM), and pH corrected to 7.2–7.4 using 5 M NaOH. Slices are placed on a membrane insert with growth media (50% Minimum Essential Media, 25% heat-inactivated horse serum, 23% EBSS, 2% B-27, and +35 mM of added glucose), and incubated at 36°C and 5% CO_2_ for 7–14 days prior to use. During recordings, slices are perfused with ACSF (in mM: 145 NaCl, 2.5 KCl, 26 NaHCO_3_, 11 glucose, 1–2 MgCl_2_ 1.2 NaH_2_PO_4_, and 2–3 CaCl_2_), bubbled with 95% O_2_ and 5% CO_2_, and heated to 32–33°C.

#### Ca^2+^ Imaging and Dye Loading

Historically, we and others have used sharp microelectrodes to simultaneously load cells with Ca^2+^ indicator dye and record from them (Emptage et al., 1999, 2003; Enoki et al., 2009). Sharp microelectrodes greatly minimize dilution of cytoplasmic contents which can otherwise impair synaptic plasticity (Malinow and Tsien, 1990; Padamsey et al., 2017). However, sharp microelectrode recordings are a challenging technique to perfect and have a lower success rate than patch microelectrodes. We therefore recommend the use of whole-cell patch recordings, which are much easier to perform.

Whole-cell patch recordings with low-resistance electrodes (4–8 MΩ) disrupt the intracellular *mileu* of cells and lead to loss of presynaptic long-term potentiation (LTP) within 10 min of break-in (Padamsey et al., 2017). In conditions where loss of plasticity is not an issue, such as in cases where only basal synaptic parameters are of interest, whole-cell recordings may be carried out with low-resistance (4–8 MΩ) patch electrodes loaded with 0.2 mM OGB-1 dissolved in standard internal solution (in mM: 135 KGluconate, 10 KCl, 10 HEPES, 2 MgCl_2_, 2 Na_2_ATP and 0.4 Na_3_GTP; pH = 7.2–7.4).

For plasticity experiments, higher resistance patch electrodes should be used (18–25 MΩ) to reduce the rate of dilution of intracellular factors. In our hands, higher resistance electrodes enable presynaptic LTP induction up to 15–20 min following break-in. These electrodes should be loaded with a higher concentration of OGB-1 (0.5–1 mM) to ensure adequate dye loading of the cell (Padamsey et al., 2017). Alternatively, our preferred method for plasticity experiments is to single-cell bolus load Ca^2+^ indicator dye into the target neuron, allowing EPSCaT imaging to proceed without electrophysiological recording or disruption of the intracellular *mileu*, and therefore without out any stringent time constraints (Padamsey et al., 2017). To perform single-cell bolus loading, we transiently patch (∼60 s) a cell with a low-resistance patch electrode (4–8 MΩ) containing a high concentration of OGB-1 (1 mM) dissolved in standard internal solution. Following loading, the patch is slowly retracted over the course of 5–10 s during which the plasma membrane rapidly reseals with very high success. We then allow 10–20 min for dye diffusion before imaging. After EPSCaT recording, which typically takes 15–20 min, the cell can be transiently re-patched to induce plasticity if required. Re-patching can be performed with very high success rates.

Regardless of the loading method, it is important that a sufficient concentration of Ca^2+^ indicator dye is present in the cell. Too little dye prevents measurement of Ca^2+^ signals with an adequate signal to noise ratio (SNR). In contrast, too much dye leads to excessive Ca^2+^ buffering within the cell, which reduces the magnitude of activity-dependent fluorescence changes, and alters the electrophysiological properties of the cell. The adequacy of loading can be assessed by triggering a back propagating action potential (bAP) and imaging fluorescence in the proximal dendrites (∼50–100 μm). We find that a resulting fractional change in fluorescence (Δ*F*/*F*) of >0.80 is indicative of a sufficient amount of dye loading in CA3 and CA1 pyramidal neurons. Dye concentration and loading times can be adjusted to achieve suitable loading levels.

For imaging Ca^2+^ fluorescence we use a BioRad MRC-1000 scan head attached to a Zeiss Axioscope upright microscope equipped with an Olympus water immersion lens (60X NA 0.90). Laser excitation can either be provided by a 488 nm solid state laser in the case of confocal imaging in cultured slices, or a Ti:Sapphire laser in the case of two photon imaging in acute slices. Emitted fluorescence is detected with a photomultiplier tube. We use LaserSharp software (BioRad) to control the microscope and acquire images, and ImageJ to analyze the images.

#### Stimulating and Searching for EPSCaTs

**FIGURE 2:**
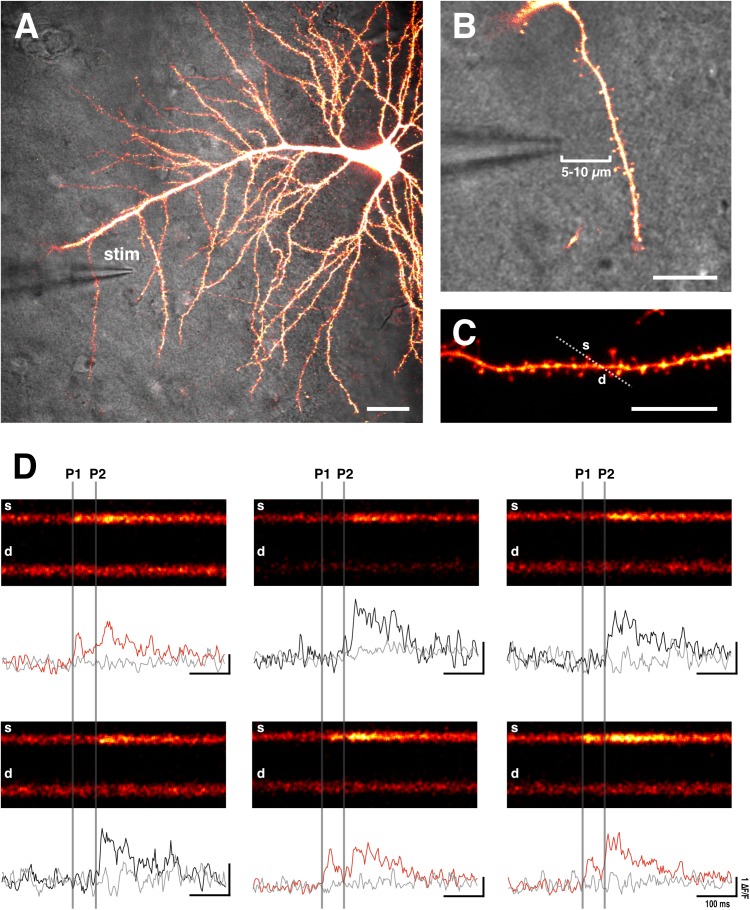
Sample EPSCaTs recordings. **(A)** Image of a CA1 pyramidal neuron that has been bolus loaded with Ca^2+^ indicator dye (OGB-1). A glass stimulating electrode (stim) is placed within 5–10 μm of a target dendrite (scale bar: 20 μm). **(B)** Magnified image of the stimulating electrode and target dendrite (scale bar: 10 μm). **(C)** Laser scanning is restricted through a line across the target spine (s) and underlying dendrite (d) (scale bar: 10 μm). **(D)** Sample line scans in which paired pulse stimulation (two pulses 70 ms apart: P1 and P2, which are denoted by vertical gray bars) is delivered following a baseline period. Raw fluorescence is quantified (Δ*F*/*F*) for both the spine (black and red traces) and dendrite (gray trace) below each line scan. Red traces show significant increases in spine fluorescence (i.e., EPSCaTs) in response to the first of the two pulses (P1); black traces fail to show spine fluorescence changes in response to P1 that are significantly different than noise. As is evident, EPSCaTs are probabilistic, restricted to the spine head, and are easily identified visually. EPSCaT probability is greater for the second of two pulses (P2), reflecting short-term facilitation. *p_r_* is calculated as the EPSCaT probability for the first pulse (P1).

**FIGURE 3:**
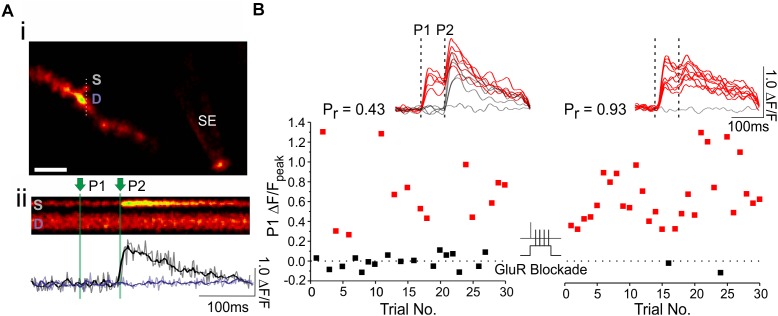
Using optical quantal analysis to image presynaptic plasticity. **(Ai)** Image of a CA1 dendrite loaded with OGB-1 (white scale bar: 2 μm). A fluorescently coated glass stimulating electrode (SE) is positioned within 5–10 μm of the imaged dendrite, and an EPSCaT-generating spine is found. Laser scanning is restricted through the spine head (S) and the underlying dendrite (D). **(Aii)** Example line scan during paired pulse stimulation in which two stimuli (P1 and P2) are delivered 70 ms apart. The quantified, smoothed fluorescent transient (Δ*F*/*F*) in the spine (black trace) and dendrite (purple trace) is shown below. **(B)** The spine is imaged during 30 stimulation trials at baseline, and another 30 stimulation trials 30 min after plasticity induction. The peak EPSCaT amplitude during the first pulse (P1) of each stimulation trial is plotted in the graph. Red points denote successful release events, in which EPSCaT amplitudes are significantly greater than noise; black points denote failures. Sample smoothed fluorescent transients from the stimulation trials are shown above, for both the baseline and post-induction periods, along with the estimates of *p_r_*. In this experiment, plasticity was induced by Hebbian stimulation, consisting of pairing single presynaptic stimuli with postsynaptic complex spikes (60 pairings repeated at 5 Hz; see text for further details). Pairing was delivered in a glutamate receptor blockade (GluR Blockade: 100 μM D-AP5, 10 μM NBQX, 500 μM R,S-MCPG, and 10 μM LY341495) designed to block all glutamate receptors. After pairing, the blockade was washed out and EPSCaTs were imaged 30 min post-induction. *p_r_* increased following paired stimulation. The experiment shows that the induction of presynaptic plasticity does not require glutamate signaling. Figure adapted from Padamsey et al. (2017) under the terms of the Creative Commons Attribution License (CC BY).

**FIGURE 4:**
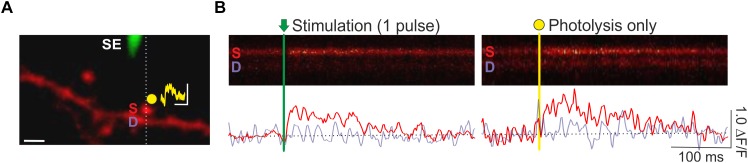
Combined optical quantal analysis with glutamate photolysis. **(A)** Image of a CA1 dendrite loaded with OGB-1 (white scale bar: 2 μm). A fluorescently coated glass stimulating electrode (SE) is positioned within 5–10 μm of an imaged dendrite, and an EPSCaT-generating spine is found. The spine is then targeted for glutamate photolysis (yellow spot). Photolysis laser power is adjusted to trigger a Ca^2+^ transient of similar amplitude and dynamics as stimulation. The resulting synaptic potential evoked by glutamate photolysis is shown above the spine head (yellow trace; scale bar: 1 mV by 100 ms). To image Ca^2+^ responses, laser scanning is restricted to a line across the imaged spine (S) and underlying dendrite (D). **(B)** Sample line scans during stimulation and glutamate photolysis. Fluorescent changes in the spine (red trace) and dendrite (purple trace) are quantified as Δ*F*/*F* in the smoothed traces below each line scan. Ca^2+^ transients evoked by stimulation and by photolysis are similar. Figure adapted from Padamsey et al. (2017) under the terms of the Creative Commons Attribution License (CC BY).

For extracellular stimulation we use a glass electrode, comprising of a low-resistance patch pipette (4–8 MΩ) filled with ACSF. A tungsten electrode, which is connected to a constant current stimulator (e.g., Digitimer) (Padamsey et al., 2017), is inserted into the pipette. The tip of the glass can be coated with bovine serum albumin fluorescent conjugate (e.g., 0.05% bovine serum albumin-Alexa Fluor 488 dissolved in 0.1 M PBS with added 3 mM NaN_3_ to maintain sterility) to aid visualization of the electrode during fluorescent imaging (Ishikawa et al., 2010; Padamsey et al., 2017). The electrode is then positioned close to an imaged dendritic branch (5–10 μm) (Yasuda et al., 2004; Padamsey et al., 2017) (Figure 2– 4). With this method, spines on the target branch have a high likelihood of responding to electrical stimulation. The dendritic branch can then be rapidly and efficiently searched during stimulation for responsive spines by using line scans (xt) that traverse as many spines as possible. During a line scan we deliver two stimulation pulses (100 μs duration) 70 ms apart in order to increase the likelihood of glutamate release via paired pulse facilitation. This is important to increase the likelihood of finding low *p_r_* synapses, and therefore to prevent selection bias in favor of high *p_r_* synapses. Stimulation intensity should be kept subthreshold for dendritic or somatic spiking, which will be evident during Ca^2+^ imaging. An EPSCaT, when triggered in the absence of dendritic or somatic spiking, should be restricted to the spine head; though some elevation in the dendrite may occur due to diffusion (Noguchi et al., 2005). The described technique typically allows 1–2 EPSCaT-generating spines to be rapidly found (1–2 min). If no responsive spines are found, the electrode can be moved a few microns, and the dendrite can be searched again. Once an EPSCaT is found, the stimulation intensity should be continually decreased until the probability of eliciting an EPSCaT is 0, after which the stimulation intensity should be increased by at least 20% to ensure that stimulation is suprathreshold for EPSCaT generation, and that any EPSCaT failures cannot be attributed to axonal stimulation failures (Emptage et al., 1999). If the EPSCaT cannot be stimulated by at least 20% above threshold intensity without eliciting a dendritic or somatic spike, then another spine should be identified.

Bringing the stimulating electrode closer than 5 μm to the imaged spine risks direct depolarization of voltage-gated channels in the dendrite or the associated presynaptic terminal. Direct depolarization of the dendrite typically results in branch-wide Ca^2+^ influx, whereas direct depolarization of the presynaptic terminal triggers spine-restricted EPSCaTs with highly inflated *p_r_* measures (unpublished observations from our lab), presumably due to elevated Ca^2+^ influx in the terminal. Indeed, studies in which stimulating electrodes are <5 μm to the imaged synapse report higher basal *p_r_* values (0.80) (Chalifoux and Carter, 2010) than those in which the electrodes are placed further from the spine (∼0.2–0.4) (Emptage et al., 2003; Ward et al., 2006; Enoki et al., 2009; Padamsey et al., 2017).

In previous studies, we and others have placed our stimulating electrode much further from the dendritic tree (>50 μm) (Emptage et al., 1999, 2003; Ward et al., 2006; Enoki et al., 2009). However, we find that with this technique it is typically more difficult to find EPSCaTs, since larger regions of the dendritic tree need to be searched for responsive spines.

For imaging EPSCaTs at postsynaptically silent synapses the Mg^2+^ block of NMDAR must be minimized to unmask EPSCaTs during synaptic stimulation. This can be achieved by either holding the postsynaptic neuron between −20 and 20 mV, or by removing extracellular Mg^2+^ from the bath solution (Ward et al., 2006).

#### Estimating *p_r_*

Once an EPSCaT-generating spine has been identified, the response of the spine to repeated trials of electrical stimulation is imaged in order to accurately calculate *p_r_*. During this time, the position of the stimulating electrode should be monitored carefully to ensure mechanical drift, which can affect EPSCaT probability, is minimal. Images are acquired as line scans (xt) through the spine and underlying dendrite (Figure 2, 3). This enables rapid acquisition of frames (500 Hz) while minimizing photobleaching. Simultaneous imaging of the spine and dendrite is important for distinguishing bona fide EPSCaTs from Ca^2+^ influx associated with local dendritic spikes or bAPs. For a given imaging trial, we typically acquire 200 successive lines at 500 Hz, for a total of 400 ms of imaging; though this will vary depending on the experiment. Single or paired pulse stimulation (70 ms interstimulus interval) is delivered 50–200 ms following the start of the scan, to enable sufficient time for baseline imaging. We prefer to use paired pulse stimulation as it makes it easier to monitor the quality and presence of EPSCaTs throughout the experiment, especially at low *p_r_* synapses. If paired pulses are used, *p_r_* is only ever calculated on the basis of the Ca^2+^ influx associated with the first of the two pulses.

Several imaging trials are required to accurately estimate EPSCaT probability, and thus *p_r_*. However, it is important to minimize imaging to minimize phototoxicity and indicator bleaching. We highly recommend including the antioxidants Trolox (1 mM) and ascorbic acid (0.2 mM) in the ACSF to help preserve the health of the spine and dendrite during imaging. When photodynamic damage is sufficient to compromise the integrity of the membrane, the dendritic compartment will rapidly brighten, and eventually bleb. A reliable estimate of *p_r_* typically requires 20–30 imaging trials, though this will depend on the actual value of *p_r_*. From the binomial theorem, the standard error (SE), and therefore the uncertainty associated with a measure of *p_r_* is:

$$\text{SE}=\sqrt{\left[\left(1-p_{r}\right)\left(p_{r}\right)/N_{\text{trials}}\right]}$$

where *N*_trials_ is the total number of imaging trials. Note the SE is minimal when *p_r_* is 0 or 1, and maximal as *p_r_* approaches 0.5. Because of this, we often have at least 30 imaging trials when imaging synapses with *p_r_*∼0.5, and 20 trials, when synapses have a *p_r_* of approximately <0.2 or >0.8; rough estimates of *p_r_* for these purposes can be derived online, during image acquisition.

Following imaging, *p_r_* is formally calculated offline as the proportion of imaging trials in which the spine exhibited a significant and selective increase in fluorescence in response to electrical stimulation. Fluorescence is calculated as:

$$\Delta F/F=(F-F_{\text{baseline}})/(F_{\text{baseline}}-F_{\text{background}})$$

where *F* is the fluorescence at any given point in time, *F*_baseline_ is the mean fluorescence at baseline, prior to stimulation, and *F*_background_ is the mean fluorescence of the background associated with regions of the image devoid of fluorescent structures. To calculate the Δ*F*/*F* associated with the putative Ca^2+^ transient (i.e., Δ*F*/*F*_transient_), we average the Δ*F*/*F* over a 50 ms time window starting from the point of stimulation. For a transient to be considered a successful release event, we require its Δ*F*/*F*_transient_ to be at least 2.5 times greater than noise, which is measured as the SE of the Δ*F*/*F* calculated during the last 50 ms of baseline imaging. The total proportion of trials in which the Δ*F*/*F*_transient_ is significantly greater than baseline noise is taken as a measure of *p_r_*.

If EPSCaT recordings are contaminated by dendritic spikes or bAPs, then it is imperative to characterize Δ*F*/*F*_transient_ for both the dendrite and spine. Dendritic spikes and bAPs generate synchronous Ca^2+^ events of similar amplitude and kinetics in both spine and dendrite, provided that both compartments are in the same plane of focus and have baseline fluorescences that are clearly distinguished from background. Consequently, EPSCaTs that co-occur with dendritc spikes or bAPs result in higher fluorescent levels in the spine than in the dendrite. In this case, a successful trial would require the Δ*F*/*F*_spine_ significantly exceed the Δ*F*/*F*_dendrite_ (Nevian and Helmchen, 2007).

### Extensions and Applications of Optical Quantal Analysis

Here, we describe extensions of optical quantal analysis, including how it can be used (1) to assess activity-dependent changes in *p_r_*, (2) to examine the impact of local synaptic signaling on *p_r_*.

#### Assessing Activity-Dependent Changes in *p_r_*

Optical quantal analysis can be conducted before and after plasticity protocols to examine activity-dependent changes in *p_r_* (Figure 3). Several protocols can be used to induce long-term changes in synaptic efficacy, though not all protocols induce presynaptic changes (Padamsey and Emptage, 2014). Presynaptic LTP induction typically requires greater levels of postsynaptic depolarization than postsynaptic LTP induction. This is because presynaptic LTP is driven by L-type voltage gated Ca^2+^ channels (L-VGCCs), which have higher voltage activation thresholds than postsynaptic NMDARs, which instead drive postsynaptic LTP (Padamsey and Emptage, 2014; Padamsey et al., 2017).

To this end, we have used several protocols successfully to induce presynaptic LTP at Schaffer-collateral synapses.

(i) The first protocol uses high-frequency stimulation (HFS) consisting of three bursts of 20 presynaptic pulses at 100 Hz, delivered 1.5 s apart. Critically, during stimulation, the postsynaptic cell should be sufficiently depolarized (5–10 mV) by current injection to ensure that the presynaptic stimulation evokes APs (Emptage et al., 2003;Enoki et al., 2009).
(ii) A spike-timing dependent plasticity (STDP) protocol can also be used. Indeed, we have recently found that pairing presynaptic stimuli with postsynaptic complex spikes provides a robust way of driving presynaptic LTP (Padamsey et al., 2017). Complex spikes are triggered by a 2–3 nA postsynaptic current injection with a 7–10 ms rising phase, a 20 ms plateau phase, and a 30–33 ms falling phase in order to emulate the kinetics of complex spikes recorded *in vivo* (Grienberger et al., 2014). During pairing, a complex spike follows the evoked presynaptic stimulus by 7–10 ms. Complex spikes can also be evoked by a conventional current injection (square pulse: 2–3 nA for 100 ms); however, spike timings are less reliable with this method. Pairing is performed 60 times at 5 Hz.
(iii) Alternatively, Enoki et al. (2009) have induced presynaptic LTP using a STDP protocol in which single presynaptic stimuli are paired with a standard burst of postsynaptic spikes (3 at 100 Hz), where each spike is generated by a 2–10 ms current depolarization. The first postsynaptic spikes follows the presynaptic stimulus by 10 ms. Pairing is repeated 100 times at 0.33 Hz in a GABA_A_ receptor blockade.

Presynaptic long-term depression (LTD) can also be induced at Schaffer-collateral synapses with several protocols.

(1) We have recently found that presynaptic LTD at these synapses is reliably triggered by autocrine activation of presynaptic NMDARs, driven by glutamate release (Padamsey et al., 2017). Consequently, protocols that strongly drive glutamate release in the absence of postsynaptic depolarization potently induce presynaptic LTD. For example, delivery of a pair of stimuli (5 ms apart), which emulates CA3 burst firing *in vivo* (Kowalski et al., 2016), 60 times at 5 Hz reliably depresses *p_r_*. Alternatively, 60–120 single presynaptic stimuli delivered at 5 Hz also induces presynaptic LTD, though only at high *p_r_* (>0.5) synapses. The postsynaptic neuron should be hyperpolarized (<−90 mV) in either case to prevent postsynaptic spiking (Padamsey et al., 2017).
(2) A STDP protocol can also be used to induce presynaptic LTD, which also depends on presynaptic NMDAR activation (Andrade-Talavera et al., 2016; Bouvier et al., 2018), likely driven by glial glutamate release (Min and Nevian, 2012). Here, three postsynaptic APs, each elicited by a 2–10 ms current injection, is followed (Δ*t* = 50 ms from the first postsynaptic AP) by a single presynaptic stimulus. Pairing is repeated 100 times at 0.33 Hz (Enoki et al., 2009).

#### Examining the Impact of Local Synaptic Signaling on *p_r_*

A key advantage of spine Ca^2+^ imaging is that it yields the spatial location of stimulated synapses, which enables spatially targeted manipulations of local synaptic signaling with photolytic uncaging (Figure 4). Previously, we have combined optical quantal analysis at single spines with MNI-glutamate uncaging to examine the impact of elevated glutamate release on presynaptic LTP and LTD (Padamsey et al., 2017). To do so, an EPSCaT-generating spine is first found. The associated spine head is then targeted for single or multi-photon glutamate spot photolysis. To deliver caged glutamate we use a local glass pipette (4–8 MΩ) connected to a picospritzer and placed within 100 μm of the imaged spine. The pipette is filled with 10 mM MNI-Glutamate dissolved in Tyrodes solution (in mM: 120 NaCl, 2.5 KCl, 30 glucose, 2 CaCl_2_, 1 MgCl_2_, and 25 HEPES; pH 7.2–7.5) and filter sterilized. We limit laser exposure to 1–2 ms using a TTL controlled shutter (LS6; Uniblitz). The laser power is adjusted so as to produce a Ca^2+^ transient with similar amplitude and kinetics as recorded EPSCaTs. In this way uncaging can be made to mimic uniquantal evoked glutamate release (Figure 3). Using this technique, we found that during presynaptic stimulation, artificially elevating glutamate release impaired presynaptic LTP induction, and promoted long-lasting decreases in *p_r_*. These effects were mediated by presynaptic NMDARs (Padamsey et al., 2017).

In addition to glutamate, we have also photo-released nitric oxide (NO) at EPSCaT producing synapses to evaluate its role in LTP induction. For this technique, caged NO (RuNOCl_3_; 0.5–1 mM) is bath applied prior to photolysis. We set the photolysis duration to 50 ms. To titrate laser power we use the NO-indicator DAF-FM (Invitrogen). The dye is first loaded into a cell by transiently patching it (60 s) with a patch electrode containing 250 μM DAF-FM dissolved in standard internal solution. After the dye reaches diffusional equilibrium (10 min), the soma is targeted for photolysis while line scan imaging. The intensity of photolysis is changed to produce an average Δ*F*/*F* increase of ∼0.07 (averaged across trials) in DAF-FM fluorescence, which amounts to 10 nM of NO based on the manufacturer’s supplied data. This concentration of NO has previously been shown to produce LTP at hippocampal synapses (Arancio et al., 1996). We calibrate laser power only once for a set of experiments. Once the intensity of uncaging is set, an EPSCaT producing spine can be targeted for spot photolysis. We have previously paired NO photolysis at the spine head with single presynaptic stimuli (30 pairings at 5 Hz). We found that presynaptic LTP was induced only when NO photolysis followed, but not preceded, the presynaptic stimulus by 7–10 ms. This suggests that NO signaling at the presynaptic terminal follows the same timing rules as traditional STDP (Padamsey et al., 2017).

## Discussion

### Limitations

We have described how to use optical quantal analysis to assess *p_r_* using Ca^2+^ sensitive dyes at single synapses in brain slice. This method is not without its limitations. Firstly, it is important to note that the selection of synapses for optical quantal analysis may incur a bias in favor of large spines producing large Ca^2+^ transients, which are more likely to catch the experimenter’s eye. Such spines may, for example, have larger number of AMPARs and/or have a higher likelihood of containing ER. Selection bias can be limited by careful and systematic scans of spines during the initial search procedure for EPSCaT-generating synapses. We find that systematic searches are easier when the stimulating electrode is placed in close vicinity of a dendrite, since the search is spatially limited. Consequently, the experimenter can spend more time carefully examining the spines in a small region of the cell, rather than attempting to assess large regions of dendrite for synaptic responses, as is the case when the electrode is placed at a distance (>10 μm) from the soma.

A second limitation of optical quantal analysis with Ca^2+^ indicator dyes is that the technique uses a postsynaptic measure to infer presynaptic function. This means that postsynaptic changes could in principle, by altering EPSCaT amplitude, impact the probability of EPSCaT detection. However, because of the excellent SNR provided by Ca^2+^ indicator dyes, EPSCaT amplitudes lie well above detection threshold. Indeed, we have shown that twofold increases or decreases in EPSCaT amplitude, induced by pharmacological alterations of postsynaptic NMDARs, do not affect estimates of EPSCaT probability, suggesting that assessment of presynaptic efficacy is unlikely to be confounded by postsynaptic factors, at least at CA3-CA1 synapses (Padamsey et al., 2017). Moreover, estimates of *p_r_* using Ca^2+^ indicators are consistent with estimates generated by other independent techniques, such as paired pulse ratio analysis (Padamsey et al., 2017).

The optical quantal analysis, as described here, considers a quantum to be any release event at the imaged dendritic spine. In practice, the vast majority of release events will be comprised of single vesicles, though a small proportion of events may consist of multiple vesicles, particularly at synapses with high basal release probabilities (Balaji and Ryan, 2007). In principle, multivesicular release may be quantified using Ca^2+^ imaging provided that EPSCaT amplitude scales with the number of vesicles of glutamate released; this could be verified, for example, by examining Ca^2+^ transient amplitudes evoked by varying intensities of glutamate photolysis at the imaged synapse. Nonetheless, *p_r_* as measured by conventional optical quantal analysis represents the probability that a synapse will release any neurotransmitter in response to an AP, regardless of whether it’s uni- or multivesicular in nature.

Another limitation of optical quantal analysis, as described here, is that it cannot be used to assess vesicular release probability (*p_v_*), which is, the probability a given vesicle will be released from the synapse. Calculation of *p_v_* requires knowledge of the total number of vesicles in the readily releasable pool of the synapse, in addition to the number of vesicles released per AP. Such measurements can be made using FM dyes and pHlourins in dissociated cultures (Ariel and Ryan, 2010; Ermolyuk et al., 2012).

Recent experimental evidence suggests that neurotransmitter release at a synapse is mediated by multiple, independent presynaptic release modules (Tang et al., 2016; Biederer et al., 2017). Conventional postsynaptic Ca^2+^ imaging cannot readily measure release probability at individual release modules, nor can other live-cell imaging techniques unless applied to reduced preparations (ex. dissociated cultures) where optical access is much improved (Midorikawa and Sakaba, 2015). *p_r_* measurements made with Ca^2+^ imaging in intact tissue will therefore reflect an integrated measure of release probability across all potential release modules associated with the imaged dendritic spine. As a consequence, observed changes in *p_r_* may reflect either an increase in average release probability across sites, or an addition of more release sites to the synapse. In the case of perforated synapses, in which dendritic spines form multiple synapses with independent boutons, estimates of *p_r_* at the spine may be confounded if more than one such bouton is recruited by electrical stimulation. This confound is unlikely to have a major impact on experimental results since perforated synapses comprise only a minority of central synapses [10–15% of mature CA1 synapses; (Harris and Stevens, 1989)].

### Wider Applicability of Optical Quantal Analysis

Although we have focussed on the application of optical quantal analysis at Schaffer-collateral synapses, it is important to note that the technique can and has been used to measure *p_r_* to other central synapses, including those of the neocortex (Reid et al., 2001; Koester and Johnston, 2005; Chalifoux and Carter, 2010; Kwon and Sabatini, 2011; Chun et al., 2014). Moreover, optical quantal analysis does not strictly require synthetic Ca^2+^-sensitive dyes. In principle, a number of probes that are able to report on transmitter release could be used to assess *p_r_* and presynaptic plasticity using the general technique we describe here. Such probes include fluorescent lipophilic dyes (e.g., FM dyes) and pH sensors (e.g., pHlourins) which report vesicular fusion, glutamate sensors which report cleft glutamate concentration, and voltage sensors, which like Ca^2+^ sensors, report the postsynaptic response of transmitter release. The only requirement for optical quantal analysis is that the chosen probe robustly and reliably detect single trial uniquantal glutamate release events with good SNR at the chosen synapse, and in the chosen experimental preparation. Optical quantal analysis with FM dyes and pHlourins is currently only possible in dissociated cultures, where optical access is optimal (Tokuoka and Goda, 2008; Ariel and Ryan, 2010). Voltage sensors are not currently sensitive enough to robustly detect single trial uniquantal glutamate release at individual synapses (Platisa and Pieribone, 2018). By contrast, recent improvements in genetically encoded Ca^2+^ (GCaMP6/7 variants) and glutamate sensors (iGluSnFR variants) make them particularly well suited for non-invasive optical quantal analysis in slice preparations with cell-type specificity (Chen et al., 2013; Marvin et al., 2017; Dana et al., 2018; Helassa et al., 2018; Jensen et al., 2018).

Despite the advantages afforded by genetically encoded sensors, they are not without limitations. Genetically encoded probes require the additional effort of sparsely transfecting tissue weeks before hand; Ca^2+^-sensitive proteins also have slow kinetics and run the risk of impacting cellular physiology due to long-term Ca^2+^ buffering. By contrast, synthetic Ca^2+^ indicators have faster kinetics and are easier to use. Dye loading via a patch pipette also naturally provides electrophysiological control of the postsynaptic cell, which would be required for most plasticity experiments during LTP or LTD induction. Moreover, postsynaptic access to the cell enables infusion of intracellular reagents and control over intracellular ion concentrations and voltage, which may be useful for examining the effects of cellular and receptor signaling on presynaptic function (Padamsey et al., 2017). Because of the ease and convenience of the technique, we would therefore highly recommend the use of Ca^2+^ indicator dyes for optical quantal analysis in brain slice experiments in which cell-type specificity is not strictly required.

### Future Outlook and Conclusion

Unfortunately, optical quantal analysis is not yet possible *in vivo* as the SNR of existing optical techniques does not allow for robust and reliable detection of single quanta of glutamate in the intact brain. However, *in vivo* optical quantal analysis is becoming increasingly likely with ongoing improvements in genetically encoded sensors Ca^2+^ (Dana et al., 2018), voltage (Storace et al., 2016; Yang and St-Pierre, 2016), and glutamate sensors (Marvin et al., 2017; Helassa et al., 2018; Jensen et al., 2018). These are complemented by advances in imaging methodologies such as three photon microscopy (Rowlands et al., 2017), adaptive optics (Ji, 2017), and endoscopy (Miyamoto and Murayama, 2016), including the use of multi-mode fibers (Vasquez-Lopez et al., 2018), which promise greater optical access *in vivo*. In conclusion, optical quantal analysis offers researchers a simple and effective method for assessing transmitter release and plasticity *in vitro*, with potential for future applications *in vivo*.

## Author Contributions

ZP drafted the manuscript. RT designed the figures. NE provided funding and overall guidance. All authors revised the manuscript.

## Conflict of Interest Statement

The authors declare that the research was conducted in the absence of any commercial or financial relationships that could be construed as a potential conflict of interest.
